# Whole-Genome Sequencing of the Nonproteolytic *Bacillus anthracis* V770-NP1-R Strain Reveals Multiple Mutations in Peptidase Loci

**DOI:** 10.1128/genomeA.00075-14

**Published:** 2014-02-13

**Authors:** Inbar Cohen-Gihon, Ofir Israeli, Adi Beth-Din, Haim Levy, Ofer Cohen, Avigdor Shafferman, Anat Zvi, Theodor Chitlaru

**Affiliations:** a Department of Biochemistry and Molecular Genetics, Israel Institute for Biological Research, Ness-Ziona, Israel; b Department of Infectious Diseases, Israel Institute for Biological Research, Ness-Ziona, Israel

## Abstract

We report the draft whole-genome sequence of the nonproteolytic *Bacillus anthracis* V770-NP1-R strain. Compared to those of other *B. anthracis* strains, the genome exhibits unique mutations in multiple targets potentially affecting proteolytic functions. One of these mutations is a deletion that disrupts the NprR quorum-sensing regulator of the NprA protease.

## GENOME ANNOUNCEMENT

Anthrax is a severe disease caused by inhalation or ingestion of or contact of skin abrasions with *Bacillus anthracis* spores that germinate in the host into toxin-producing vegetative bacilli, resulting in lethal massive bacteremia and toxemia. *B. anthracis* is an A-class CDC select agent, and the disease is endemic in some regions. One of the 3 toxin subunits, protective antigen (PA), constitutes the basis for all anthrax vaccines (1).

*B. anthracis* V770-NP1-R (ATCC 14185) is an acapsular strain (devoid of the native plasmid pXO2 that encodes capsid synthesis functions) selected half a century ago on the basis of its limited proteolytic activity, which enabled its extensive use for the efficient production of PA subunit vaccines (2, 3) and as a platform for the heterologous expression of recombinant antigens (4, 5). A proteomic survey of various virulent and avirulent strains allowed us to determine that ATCC 14185 bacteria fail to synthesize NprA (locus GBAA0599), the major secreted protease of *B. anthracis*, which was shown to digest PA (6–8). Here, we report the whole-genome sequence of *B. anthracis* ATCC 14185. Genomic DNA was prepared using standard methods. Sequencing was performed using an Illumina GAIIe platform (Illumina, Inc.) by generating paired-end reads with an insert size of ~300 bp. Quality-filtered reads (8,983,579 paired-end reads, 75 bp each) were assembled using Velvet (9) in conjunction with the VelvetOptimiser (http:/bioinformatics.net.au/software.velvetoptimiser.shtml). The resulting contigs were ordered against the reference *B. anthracis* Ames ancestor genome (NCBI accession no. NC_007530) using Mauve (10). The final assembly consists of 36 contigs, with an assembled genome length of 5.16 Mbp. The average coverage across the genome is 161-fold. Single-nucleotide polymorphisms (SNPs) were identified using SAMtools (11) using short-read alignment to the reference genome generated by Novoalign. Custom Perl scripts were developed to assign mismatches to open reading frames and to filter data relating to functionally annotated proteases.

Over 99.6% of the sequence was mapped to the chromosome of the reference genome, with an average coverage of 202.9-fold, a total of 601 mismatches (two-thirds of which are strain specific), and a similar G+C content (35.25%). The average coverage for the pXO1 plasmid was 539.9-fold. As expected, no pXO2 sequences were detected. We identified at least 5 unique features that may explain the nonproteolytic phenotype of the ATCC 14185 strain, including 2 indels, located within the open reading frame for proteases (loci GBAA1295 [analogous position in the reference genome, 1240740] and GBAA3785 [3476742]), as well as 2 nonsynonymous SNPs (GBAA2860 [2651101] and GBAA3660 [3368390]).

Interestingly, an inspection of the V770-NP1-R genome identified one nonsense mutation within the *nprR* gene (locus GBAA0597 [position 612228 in the reference genome]), which affects the expression of the NprR/NprX quorum-sensing regulon active in bacilli belonging to the *Bacillus cereus* group (12, 13). Therefore, this observation may explain the absence of the NprA (locus GBAA0599) protease expression evidenced by the proteomic data (6), which demonstrated downregulation of the NprA protein in spite of an intact *nprA* gene.

Overall, the availability of this genome sequence enables the identification of proteases affected in this strain, and it therefore may be useful in guiding the engineering of novel nonproteolytic *B. anthracis* strains tailored for the production of native or specific recombinant proteins.

### Nucleotide sequence accession numbers.

The whole-genome shotgun project has been deposited at DDBJ/EMBL/GenBank under the accession no. AZQO00000000. The version described in this paper is version AZQO01000000.
